# Multiturn dialogue generation by modeling sentence-level and discourse-level contexts

**DOI:** 10.1038/s41598-022-24787-1

**Published:** 2022-11-27

**Authors:** Yang Yang, Juan Cao, Yujun Wen, Pengzhou Zhang

**Affiliations:** State Key Laboratory of Media Convergence and Communication, Communication University of China, Beijing, 100024 China

**Keywords:** Computer science, Software

## Abstract

Currently, multiturn dialogue models generate human-like responses based on pretrained language models given a dialogue history. However, most existing models simply concatenate dialogue histories, which makes it difficult to maintain a high degree of consistency throughout the generated text. We speculate that this is because the encoder ignores information about the hierarchical structure between sentences. In this paper, we propose a novel multiturn dialogue generation model that captures contextual information at the sentence level and at the discourse level during the encoding process. The context semantic information is dynamically modeled through a difference-aware module. A sentence order prediction training task is also designed to learn representation by reconstructing the order of disrupted sentences with a learning-to-rank algorithm. Experiments on the multiturn dialogue dataset, DailyDialog, demonstrate that our model substantially outperforms the baseline model in terms of both automatic and human evaluation metrics, generating more fluent and informative responses than the baseline model.

## Introduction

Dialogue systems consist of task-oriented dialogue systems^1–3^ and non-task-oriented dialogue systems^4–6^, namely, open-domain dialogue systems. The former addresses concrete tasks in specific domains, including ticket booking and food ordering. A task-oriented dialogue system is easy to implement and already has many real world applications. Recently, with the availability of large dialogue datasets^7–10^ and the rapid development of pretrained models^11,12^, open-domain dialogue has yielded considerable benefits. However, the open-domain dialogue systems’ challenge is generating coherent and consistent text by effectively modeling the dialogue context.

Dialogue context is composed of system information and user information, which provides the user target and topic information of the current dialogue, and therefore, plays a crucial role in response generation^13^. Early research focused on concatenating dialogue context or inputting a fixed window size of dialogue context into the model for response generation^14,15^. The proposed hierarchical recurrent encoder–decoder (HRED) neural network was first utilised in a dialogue system^16^. It employed a two-layer RNN encoder model and built a word-level encoder to learn sentence representation in a dialogue and a high-level context encoder to track and pass on topic information from previous turns. Xing et al.^4^ added attention mechanisms to the hierarchical neural network model to help the model learn the important parts of the dialogue history. Recently, Gu et al.^17^ used a hierarchical transformer model, which proposed two learning tasks for masked sentence regression and sentence order prediction to capture the discourse-level consistency between sentences. Although the hierarchical neural network model focused on high-level semantic information at the discourse-level compared to a straightforward concatenation of dialogue contexts, it ignored dialogue history information when learning individual sentence representations, which is important for understanding current sentences.

In this paper, inspired by the DialoFlow model^18^, we introduce a context difference-awareness module to model context information on the sentence level while focusing on the semantic impact of each sentence on the whole dialogue. To leverage the relationships within sentences, we first use a bidirectional transformer encoder to obtain a dense representation of the sentences and then feed the output of the encoder into a difference-aware module that captures the difference information across the previous multiturn contexts. Finally, the response is generated in the decoder utilising the difference information and the previously decoded token. The sentence order prediction (SOP) task is also designed in the encoder to capture the discourse structure of the dialogue context. Our contribution is divided into two main aspects as follows.

1. We propose a difference-awareness module. Difference information is constructed by utilising multiturn contextual representations to dynamically capture the influence that each sentence has on the whole dialogue, and use it for target sentence generation.
2. We introduce a sentence order prediction task that converts a ranked list of dialogue contexts into a top-one probability distribution with a learnable ranking function. The disordered sentences are organised into a coherent dialogue while effectively modeling the dialogue context at the discourse level.

We conduct experiments on the multiturn dialogue dataset, DailyDialog. The results indicate that our model outperforms the baseline model on automatic evaluation metrics, such as BLEU, NIST and entropy. The human evaluation metrics further validate our perspective on our model’s ability to effectively model dialogue context and generate coherent and diverse responses.

## Related works

Related work has focused on pretrained language models, pretrained dialogue models and context-aware dialogue models.

### Pretrained language models

In recent years, pretrained language models have been rapidly developing in the field of natural language processing (NLP). The basic idea is to first train models on a large unsupervised corpus and then fine-tune these models on a downstream supervised corpus, which allows the models to achieve remarkable results. As the computing power of computers became larger and the transformer model^19^ developed, a number of pretrained language models emerged, represented by the bidirectional language model, BERT^12^, and the unidirectional autoregressive language model, GPT^11^. The BERT model can be regarded as the encoder part of the transformer model, which uses two pretraining tasks, the masked language model and the next sentence prediction to train the model. The GPT model is the decoded part of the transformer model, a generative pretrained model. GPT employs a two-stage training model, and the first stage is pretraining using a standard language model; the second stage is solving downstream tasks by fine-tuning. To obtain a more powerful model, GPT-2^20^ followed the basic network architecture of GPT using a larger dataset and more network parameters and the learned model without additional training when transferring to downstream tasks. GPT-3^21^ utilised the GPT-2 structure and reached 175 billion and 45 TB for the number of parameters and training dataset, respectively, while outperforming other fine-tuned results on many complex NLP tasks, such as machine translation. There are also improved models of BERT, such as ERNIE^22^, which introduced common sense, MASS^23^, which was improved based on the generation task, and RoBERTa^24^, which was finely tuned for parameters. Developments in pretrained language models have influenced dialogue generation. The BART model^25^ combined the bidirectional encoder of BERT with the unidirectional decoder of GPT and improved BERT’s masking strategy, which is more suitable for text generation tasks.

### Pretrained dialogue models

Because of the difficulty of obtaining large training sets of dialogue, many researchers have trained on pretrained language models. The DialoGPT^14^ model was trained on a pretrained GPT-2 using Reddit data. Meena^26^ employed more social media data and larger parameters to train a pretrained model and made considerable improvements in multiturn dialogue generation. The Blender model^27^ was the first chatbot in the world to incorporate different conversational skills. It combined the dialogue skills of empathy, knowledge and personality in a dialogue system that was more human in terms of engagement. Aiming to address the problem of dialogue response diversity, the PLATO model^15^ was the first to propose a pretrained dialogue generation model based on a latent space. Subsequently, PLATO-2^28^ adopted a course-learning approach based on the PLATO model, further expanded the network scale and increased the training data. PLATO-XL^29^ introduced a multiparty-aware input representation based on an expanded network structure and dataset to clearly distinguish individual roles in multiturn dialogue and enhance the consistency of the model over multiturn dialogue.

### Context-aware dialogue models

Both generation-based and retrieval-based dialogue systems rely heavily on dialogue contexts, and hence, effective modeling of dialogue contexts can play a substantial role in improving the quality of model generation. CSRR^30^ suggested using a dialogue semantic relation RNN to model dialogue contexts explicitly from three different levels of latent variables. The discourse level captured global information, the pair level acquired topic information between query and response pairs, and the utterance level represented content difference information. In multiturn dialogue generation, responses are generally relevant to only a few contexts, though the hierarchical encoder–decoder model uses a traditional attention mechanism to process all contextual information, which can lead to insufficient relevance. Therefore, the ReCoSa^31^ model was designed to solve this issue. A word-level LSTM encoder was used to encode the dialogue context, then a self-attentive mechanism was applied to further capture the dialogue context representation, and finally, the relationship between the context and the response was calculated by the cross-attentive mechanism of the encoder and decoder. The structural information of the dialogue was accessed in an unsupervised manner^32^, which suggested adding structured attention mechanisms to variational recurrent neural networks. Furthermore, the DialoFlow^18^ model was presented to resolve the fact that hierarchically structured dialogue models ignored dialogue contextual information when learning individual sentences. With this model, the dialogue context was modeled through a dynamic flow mechanism. Inspired by DialoFlow, our model introduces difference information among multiturn contexts and refactoring to disrupt the order of sentences, modeling dialogue history at both the sentence level and the discourse level. In contrast to the DialoFlow model, we enhance the contextual difference information and introduce an SOP learning task.

## Methodology

All experiments on human participants were carried out in accordance with the relevant guidelines and regulations of the National Key R&D Program of China (2020AAA0108700).

All experimental protocols are approved by the Ethics Committee of the Institute of Internet Information of the Communication University of China.

Written informed consent was obtained from all human participants.

### Model definition

In multiturn dialogue generation, given a dialogue context for each turn, our goal is to generate a response that is relevant to the current dialogue context. Let $u^{T} = \{ x^{T} ,y^{T} \}$ define a dialogue in the *T*-th turn, where $x^{T} = \{ x_{1}^{T} ,...,x_{{\left| {x^{T} } \right|}}^{T} \}$ is a user input and $y^{T} = (y_{1}^{T} ,...,y_{{|y^{T} |}}^{T} )$ is a response. In addition, we define the dialogue context in the *T*-th turn as $c^{T} = [x^{T - 1} ;y^{T - 1} ;x^{T} ]$.

### Model architecture

**Figure 1:**
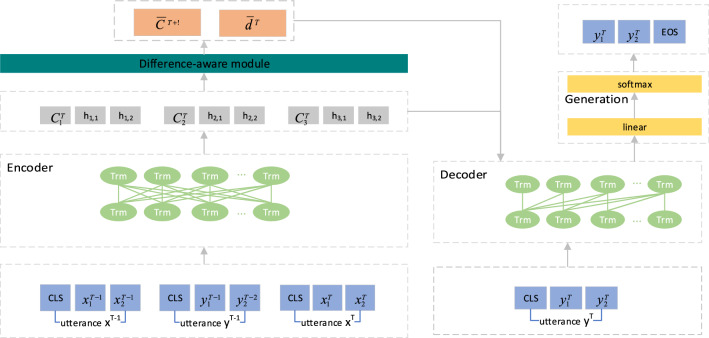
The architecture of our model. [CLS] is a special token placed at the beginning of each sentence and captures a dense representation of each sentence. For simplicity, we use two tokens to represent a sentence and omit the special token [SEP] at the end of each sentence.

Our model consists of four modules: bidirectional transformer encoder, unidirectional transformer decoder, difference-aware and response generation, where the encoder and decoder are made up of multiple transformer blocks. The overall architecture of the model is shown in Fig. 1. We first use the encoder of the BART model to encode the dialogue context $c^{T}$ of the *T*-th turn and obtain the dense context representation, $C^{T} = \{ C_{1}^{T} ,C_{2}^{T} ,C_{3}^{T} \}$. The difference-aware module is introduced to the model to compute the difference of the information between the (*T* + 1)-th turn and the previous *K* turns of context representation to dynamically capture context information. The obtained difference information and the previously decoded token are then fed into the decoder to obtain the hidden state $H^{T}$ of the decoder. Finally, in the response generation module, the target sentence $y^{T}$ is generated based on the hidden state of the decoder.

#### Input representation

**Figure 2:**
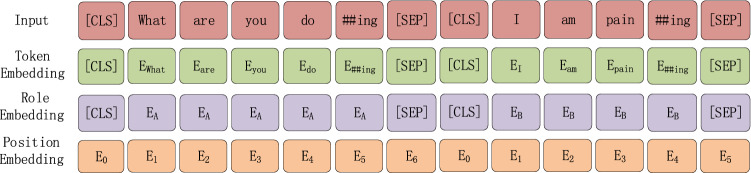
Input representation of the model.

The input embeddings of our model include the corresponding token and role and position embeddings, which are visually represented as shown in Fig. 2. The details are as follows.

- For each sentence, $u_{i} = (u_{i,1} ,u_{i,2} ,...,u_{{i,|u_{i} |}} )$, the input of our model adds [CLS] and [SEP] characters to the first and last parts of each sentence, respectively. The [CLS] character is applied to extract the representation of each sentence, while the [SEP] character is used to split the different sentences. The input text is tokenised with the WordPiece of the BERT model.
- Role embedding is applied to distinguish the identity of different speakers. In dialogue responses, we assign various roles to different speakers. Assuming there are two speakers, the first and second speakers generate tokens with role embeddings, E_A_ and E_B_, respectively. The positional embedding is obtained through the position where the token is located in each utterance. Ultimately, the input embeddings are the sum of the corresponding token, role and position embeddings.

#### Difference-aware module

We employ a layer of transformer blocks, take the context representation of the previous *T* turns as input, predict the (*T* + 1)-th turn context representation, and compute the difference at the (*T* + 1)-th turn context representation $\overline{C}^{T + 1}$ and the previous *K* turns context representation $\{ C^{T - k} \}_{k = 0}^{K}$ denoted as $\overline{d}^{T}$:

1 $$\overline{C}^{T + 1} = transformer(C^{1} ,C^{2} ,...C^{T} )$$

2 $$\overline{d}^{T} = \sum\limits_{k = 0}^{K} {\lambda_{k} } MLP([\overline{C}^{T + 1} ;C^{T - k} ;\overline{C}^{T + 1} - C^{T - k} ])$$

3 $$\lambda_{i} = \frac{{{\text{exp}}(\overline{C}^{T + 1} W_{1} C^{T - i} )}}{{\sum\nolimits_{k = 0}^{K} {\exp (\overline{C}^{T + 1} W_{1} C^{T - k} )} }}$$

where $\lambda_{k}$ is a weight, *MLP* is a fully connected layer activated by tanh, $W_{1}$ is a trainable parameter, and $K$ is a hyperparameter. Note that in the first turn, we set $d^{1}$ to be a zero vector, since different information cannot be obtained in the current turn.

#### Response generation module

The response generation module consists of a linear layer and a softmax layer. As depicted on the right in Fig. 1, we take the context representation $C^{T}$, the output $\overline{d}^{T}$ of the difference-aware module and the previously decoded token as input and feed them into the transformer decoder to obtain the output $H^{T}$ of the decoder in the current turn. In generating the response $y_{i}^{T}$, we estimate the probability distribution of the *i*-th token given $H^{T}$ as follows:

4 $$p(y_{i}^{T} |y_{ < i}^{T} ,u_{N}^{T} ) = soft\max (W_{2} H^{T} + b_{1} )$$

where $W_{2}$ and $b_{1}$ are trainable parameters. Hence, the objective of response generation is defined as follows:

5 $${\text{L}}_{{{\text{gen}}}} = - \sum\limits_{m = 1}^{n} {\log p(y_{i}^{T} |y_{ < i}^{T} ,u_{N}^{T} )}$$

### Training tasks

#### Sentence order prediction (SOP)

**Figure 3:**
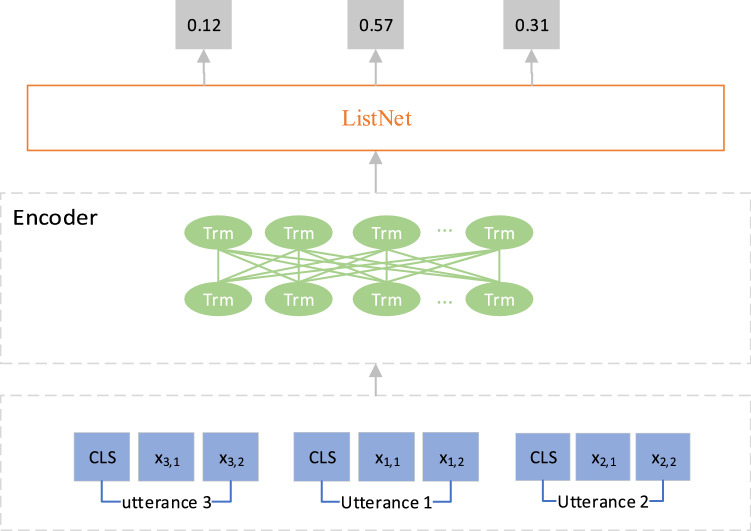
Sentence order prediction task.

The SOP task is to reconstruct the randomly disrupted dialogue context order by implementing a sentence reordering algorithm. That is, given a randomly disordered context $$\mathrm{C}=\{{\mathrm{u}}_{\mathrm{s}1},{\mathrm{u}}_{\mathrm{s}2},...{\mathrm{u}}_{\mathrm{sm}}\}$$ with order $$s = [s1,s2,...sm]$$, we use the learning-to-rank (L2R)^33^ algorithm to obtain a coherent sequence of contexts $$\widetilde{C} = \left\{ {u_{{\overset{\lower0.5em\hbox{$\smash{\scriptscriptstyle\smile}$}}{s}_{1} }} ,u_{{\overset{\lower0.5em\hbox{$\smash{\scriptscriptstyle\smile}$}}{s}_{2} }} , \ldots u_{{\overset{\lower0.5em\hbox{$\smash{\scriptscriptstyle\smile}$}}{s}_{m} }} } \right\}$$ and its order $$\widetilde{s}=[\widetilde{s1},\widetilde{s2},...,\widetilde{sm}]$$. As illustrated in Fig. 3, it is assumed that our context consists of three sentences in a disordered order, and they are fed into the encoder to obtain the hidden state of each sentence. The probability distribution of these sentences is then returned as [0.12, 0.57, 0.31] by a ranking learning algorithm, and the order s = [1, 2, 0]. Note that in order for the model to see a natural sequence of inputs, we disorder sixty percent of the contexts.

The goal of L2R is to apply machine learning techniques to learn the ranking function, which obtains the relevance score between all document sequences and the current query and ranks the document sequences based on the score. In the training phase, we apply ListNet^33^ algorithm to reconstruct the order of disrupted sentences. A global attention mechanism^34^ is applied to estimate the contribution $f(h^{(i)} )$ of each sentence *i* to the overall dialogue. The top-one probability of each sentence is then calculated with softmax, which represents the probability of every sentence being ranked in the first place in the current context. The details are given in Eqs. (6) and (7) as follows:

6 $$f(h^{(i)} ) = \frac{1}{|m|}\sum\limits_{j = 1}^{|m|} {R_{i}^{T} WR_{j} }$$

7 $$p_{{(u_{i} )}} = {{\varphi (f(h^{(i)} ))} \mathord{\left/ {\vphantom {{\varphi (f(h^{(i)} ))} {\sum\nolimits_{k = 1}^{|m|} {\varphi (f(h^{(k)} ))} }}} \right. \kern-\nulldelimiterspace} {\sum\nolimits_{k = 1}^{|m|} {\varphi (f(h^{(k)} ))} }}$$

where $\varphi ( \cdot )$ is the exponential function, and $R_{i}$ and $R_{j}$ denote the hidden states of the *i*-th and *j-*th sentence encoders, namely, the output of the corresponding special token [CLS]. The top-one probability $q(u{}_{i})$ of the golden target sentence is defined as follows:

8 $$q(u_{i} ) = {{\varphi (y_{i} )} \mathord{\left/ {\vphantom {{\varphi (y_{i} )} {\sum\nolimits_{k = 1}^{|m|} {\varphi (y_{k} )} }}} \right. \kern-\nulldelimiterspace} {\sum\nolimits_{k = 1}^{|m|} {\varphi (y_{k} )} }}$$

9 $$y_{i} = \frac{{|m| - s_{i} }}{|m|}$$

where $y_{i}$ is the golden score for each sentence $u_{i}$. $s_{i}$ is the golden order of the sentences. Note that the order of the sentences starts from 0.

We finally measure the distance between the golden distribution and the training distribution using the cross-entropy as follows:

10 $$L_{lr} = - \sum\limits_{i = 1}^{m} {q(u_{i} )} \log p(u_{i} )$$

#### Total training objective

The overall training objective of our model can be expressed as follows:

11 $$L = L_{gen} + \lambda_{1} L_{lr} + \lambda_{2} L_{sim}$$

12 $$L_{sim} = \sqrt {\sum\limits_{k = 1}^{n} {|\overline{C}^{K} - C^{K} |} }$$

where $\lambda_{1}$ and $\lambda_{2}$ are adjustable parameters and $L_{sim}$ is a context similarity modeling task^18^. It minimises the Euclidean distance between the predicted context and the true context by setting the predicted context in the difference-aware module increasingly close to the true context.

By pretraining with sentence order prediction and contextual similarity tasks, the encoder is able to better capture contextual discourse structure and semantic information. In the fine-tuning process, we train our model only with the response generation objective.

## Experiments

### Experimental dataset

We evaluate our model on the DailyDialog^9^ dialogue dataset. DailyDialog is a multiturn dialogue dataset for daily chat scenarios that is written by English learners, and thus, is more grammatically rigorous, has less noise, and focuses more on various life topics. There are a total of 13,118 conversations, each with approximately 8 turns. We divided the dataset into a training set (80%), a validation set (10%) and a test set (10%). The statistics of the dataset are shown in Table 1.

**Table 1:** Statistical results on the DailyDialog dataset.

Training set	11,118
Validation set	1000
Test set	1000
Total dialogues	13,118
Average speaker turns per dialogue	7.9
Average tokens per dialogue	114.7
Average tokens per utterance	14.6

### Implementation details

We take the BART model as the basic framework for our model, as it has been widely used in text generation models. We follow the hyperparameters of the BART-large model and adopt pretrained checkpoints to initialise our model. In the BART-large model, both the encoder and decoder have 12 layers, the hidden state is 1024 dimensions, and there are 16 heads, each with 64 dimensions. In addition, the size of the batch is 32, the maximum length of both the encoder and decoder is limited to 128, and the maximum number of sentences in each context is 7. We empirically set both the adjustable factors $\lambda_{1}$ and $\lambda_{2}$ in Eq. (11) to 1. Our model is optimised by Adam^35^, which has an initial learning rate of 5e − 5, and we optimise the learning rate using a warm-up strategy with an initial warm-up step of 5000. In the response generation phase, we use a beam search for decoding with a beam size of 4. We set *K* to 1 when solving for difference information.

To make the experimental data more convincing, we repeated the experiment five times and finally displayed the average results of the experiment. We conducted the experiments on a NVidia Tesla K80 with 12 GB of memory.

### Baselines

The Seq2Seq model utilises a transformer encoder-decoder architecture to train the model from scratch on a downstream dataset with a language modeling task.

The GPT-2^20^ model is a left-to-right transformer decoder architecture. It trains an encyclopaedia-like model with a 40 GB ultra-large dataset and large model parameters that can be applied to various generative tasks without fine-tuning.

The BART^25^ model is a standard seq2seq architecture with a bidirectional encoder and a left-to-right decoder. BART is well-suited for fine-tuning text generation tasks but is also appropriate for text comprehension tasks.

The DialoGPT^14^ model is an open-domain dialogue system that employs a causal language modeling (CLM) task to train the model on large-scale dialogue data. A maximum mutual information (MMI) scoring function is designed to penalise safe responses.

The DialoFlow^18^ model constructs a dialogue flow model using the pretrained GPT-2, which introduces a dynamic flow mechanism to model the dialogue history and proposes three training objectives, contextual flow modeling, semantic influence modeling and response generation modeling to optimise the model.

### Evaluation metrics

#### Automatic evaluation

We use the evaluation metrics, BLEU^36^ (B-n), NIST^37^ (N-n), Entropy^38^ and lexical repetition (LR-n)^39^, which are commonly used in language models as our automatic evaluation metrics. BLEU was previously used as a tool to evaluate the quality of machine translation. The quality of a candidate text is measured by calculating the overlap of n-grams between the candidate text and the reference text. NIST is a variation of BLEU that introduces the amount of information in each n-gram and enlarges the weight of words with few but important occurrences. LR-n is employed to calculate the percentage of texts with 4-g that are repeated at least n times in all generated texts, and we set n to 2. BLEU and NISTL are applied to measure the correlation between the reference text and the generated text, while the entropy evaluation metric is used to estimate the diversity of the generated vocabulary.

#### Human evaluation

We follow previous work^26^ to measure our model using specificity, sensibleness, and the average of specificity and sensibleness (SSA). These three metrics range from 0 to 1. Specificity measures whether the generated text is diverse and unique, while sensibleness measures the accuracy and the fluency of the generated text. The specificity metric is also set to 0 if the sensibleness metric is 0.

### Experimental results

We conducted experiments on the DailyDialog dataset with automatic evaluation metrics and human evaluation metrics. In addition, we verified the effect of various difference operations on the model. To intuitively reflect the generative effect of our models, we provide some generative examples.

#### Automatic evaluation

Table 2 shows the automatic evaluation metrics for our model and the baseline model on the DailyDialog test set. All models in the experiments employ a 12-layer transformer block and use a beam search for decoding. We also conducted ablation experiments to demonstrate the effectiveness of the proposed training task. Our model achieves the best results on most automatic evaluation metrics by exploiting contextual difference information and pretraining on the proposed training task. Compared with models that statically model contextual information, such as GPT-2, BART and DialoGPT, our model outperforms the baseline model on the relevance evaluation metrics, BLEU and NIST, which indicates that our model generates text with higher word overlap with the reference text. As a result, our model is better at capturing semantic features and tends to generate high-quality target text. Furthermore, our approach does not compromise the diversity of responses. Our model is also comparable to the best baseline models in terms of entropy, and our model yields informative texts. The ablation experiments revealed that SOP and CSM are quite important for generating relevant text and that they are also beneficial for reducing lexical repetition. When we train the model using only the SOP task, all of the model’s metrics are reduced substantially, reflecting the importance of contextual similarity modeling to capture contextual consistency.

**Table 2:** Results of automatic evaluation metrics on the DailyDialog test set. Statistical significance of the differences is calculated with a two-tailed t-test. w/o SOP indicates that the model ablates the sentence order prediction task, w/o CSM indicates that the model ablates the contextual similarity modeling task, and Flow w/ aux indicates that the DialoFlow model is fine-tuned on the SOP and CSM training tasks. *p < 0.05, comparison with ours.

Model	B-2	B-4	N-2	N-4	Entropy	LR-2
Seq2Seq	17.68*	5.45*	1.44*	1.59*	7.61*	26.0*
GPT-2	17.96*	5.87*	1.86*	1.95*	8.12*	31.2*
BART	18.21*	5.99*	1.68*	1.83*	8.35*	28.5*
DialoGPT	18.83*	6.63*	2.29*	2.78*	9.20	30.5*
DialoFlow	26.47	10.12	3.65*	3.84*	9.82*	26.4*
Flow w/ aux	**27.35**	10.45*	3.77	3.99*	9.77	25.7
Ours	27.03	**11.37**	**4.05**	**4.33**	**9.89**	25.3
w/o SOP	27.18	11.05	3.93	4.02	9.82	**25.0**
w/o CSM	26.36	10.40*	3.54	3.80	9.35	25.8

The best experimental results are in [bold].

During the generation phase, both our model and the DialoFlow model employ contextual difference information. However, our model focuses not only on the difference information of previous K-turns but also on its own information, which can enhance the quality of text generation. To validate the effectiveness of our proposed difference-aware module, the DialoFlow model is fine-tuned on the DailyDialog dataset with the proposed training target as an auxiliary task (Flow w/ aux), and the experimental results are shown in Table 2. Compared with our model, the DialoFlow model with fine-tuning is lower than our model in all evaluation metrics except for the BLEU-2 metrics.

#### Human evaluation

**Figure 4:**
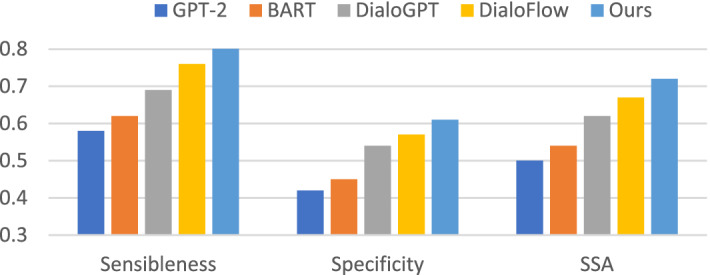
Human general evaluation results

As automatic evaluation metrics do not fully reflect the quality of the generated text, we apply human evaluation metrics. We first sample 300 random dialogues on the DailyDialog testset, where each dialogue is adjudicated by four annotators. The annotators are all well-educated native speakers and have received online training. Statistical annotator agreement. For sensibleness, the probability of at least three annotators giving the same label (3/4 agreement) is 83.5%, and the probability of four annotators giving the same label (4/4 agreement) is 48%. The results of the experiments are presented in Fig. 4. We randomly selected three samples and their human evaluation scores are shown in Table 3. We can see that our model achieves a higher sensibleness and specificity than the baseline model. This illustrates that our model can produce informative responses while maintaining fluency. The GPT-2 and BART models tend to generate safer responses, such as "I do not know", which is generally fluent, and therefore, these two models obtain lower specificity among all of the models.

**Table 3:** Human evaluation score on the DailyDialog test set.

Context	GPT-2	BART	DialoGPT	DialoFlow	Ours
A: My dear, what's for supper? B: Red cooked carp and rape with fresh mushrooms A: What about soup? B: Sour-peppery soup	Thanks. this is bad bad [Sensible: 0 Specific: 0]	Do you need some food? [Sensible: 0 Specific: 0]	Yes, me too. I am making this soup [Sensible: 0 Specific: 0]	This soup is delicious, I love it [Sensible: 1 Specific: 0]	I want to know which condiment you use [Sensible: 1 Specific: 1]
A: Good evening, madam. Can I help you? B: Yes. I bought this sheet here yesterday A: Anything wrong with it? B: Yes. When I spread it out back home, I found this hole	Thank, only need more [Sensible: 0 Specific: 0]	Then what happened [Sensible: 1 Specific: 0]	Well, Let me see, I'm terribly sorry [Sensible: 1 Specific: 0]	I'm sorry, What I can do for you [Sensible: 1 Specific: 0]	Oh, yeah, it’s terrible. May I know your order? [Sensible: 1 Specific: 1]
A: Good afternoon! Can I help you? B: Could you show me where the Chinese-style clothing is located? I want to buy a silk coat A: This way, please. Here they are. They're all handmade B: Oh, they look nice. May I try that black one on?	Sure, no problem [Sensible: 1 Specific: 0]	Please. It is wonderful [Sensible: 0 Specific: 0]	Black for you [Sensible: 0 Specific: 0]	Yes, of course, this way please [Sensible: 1 Specific: 1]	Sure, the fitting room is on your right [Sensible: 1 Specific: 1]

**Figure 5:**
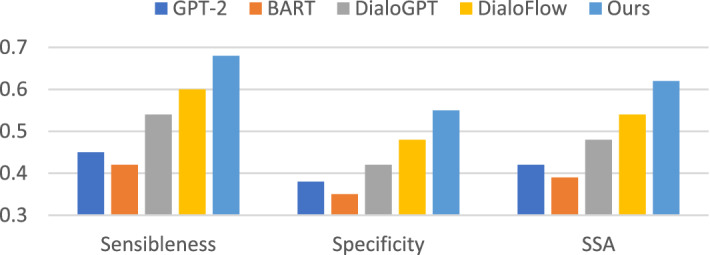
Human interactive evaluation results.

We also perform an interactive human evaluation using the three evaluation metrics described above to restore a realistic scenario of human–machine dialogue. For each model, we ask participants to engage in at least six turns of dialogue with the robot and score each sentence according to the human evaluation metrics. We employ the same annotators as for the general human evaluation. For specificity, the probability of at least 3/4 agreement is 82.9% and 4/4 agreement is 49.1%. The evaluation results are displayed in Fig. 5. It can be observed that in real scenarios, our model sensibleness scores improve more substantially than the other baseline models compared to sampling on the test set. This demonstrates the superior generative ability of our model in the multiturn of human–machine interaction.

#### Difference information

We prove the effectiveness of our model. In this section, we apply various difference operations on the DailyDialog dataset to better understand the impact on the model. The BLEU results are reported in Table 4. For simplicity, we only use the contextual information from the previous turns when calculating the difference information.

**Table 4:** BLEU results for difference operations.

Difference	BLEU-2	BLEU-4
$(m;n)$	20.12	6.05
$(|m - n|)$	24.19	9.31
$(m * n)$	24.64	9.86
$(m;n;m * n)$	25.29	10.15
$(|m - n|;m * n)$	25.70	10.78
$(m;n;|m - n|)$	**27.03**	**11.37**
$(m,n,|m - n|;m * n)$	26.85	11.02

The best experimental results are in [bold].

As seen from Table 4, BLEU-2 and BLEU-4 have the lowest scores when employing only contextual information in the model at 20.12 and 6.05, respectively, and the BLEU scores improve remarkably with the addition of the difference information. This further supports our view that in addition to taking advantage of the contextual information in the generation phase, the difference information is more likely to improve the quality of the generated text. In addition, we note that adding elementwise difference information $m * n$ degrades the model’s performance. The most important of these difference operations is the elementwise difference $|m - n|$, which measures the distance between two sentence representations, ensuring that similar contexts are closer and that dissimilar contexts are further apart.

#### Case study

We provide generated samples from the DailyDialog testset in Table 3. The generated results show that our model is able to produce more coherent and relevant responses than the baseline model. This is clearly consistent with the results of our automatic and human evaluations. Moreover, we note that the GPT-2 model performs worse than the other baseline models, which generate a context-independent response. This can be attributed to the fact that it adopts a unidirectional autoregressive model, which prevents it from performing well across multiturn dialogues. DialoGPT introduces maximum mutual information on the basis of GPT-2 to penalise hypotheses that are frequent and repetitive, resulting in a more diverse set of responses. The DialoFlow model adds difference information to the GPT-2 model to generate more contextually relevant responses than the DialoGPT model. In contrast, our model applies the bidirectional model, BART, and introduces difference information and auxiliary tasks to generate responses that are closer to human responses in multiturn dialogues.

## Conclusion

In this paper, we propose a multiturn dialogue generation model to dynamically model dialogue contexts. Specifically, we apply a difference-awareness module to capture the semantic impact on the whole dialogue through every sentence. A sentence order prediction training task is also designed to learn the discourse structure of the dialogue context by reconstructing the order of the disrupted sentences. We perform experiments on the multiturn dialogue dataset, DailyDialog. Our model achieves notable performance on relevance and diversity automatic evaluation metrics. The human evaluation metrics further demonstrate of our model’s ability to produce fluent and consistent text without sacrificing diversity. Additionally, we perform difference-information experiments to verify the impact of various difference operations on the model. In the future, we hope to apply difference information to other text generation tasks, such as data-to-text generation.

## Data Availability

All data included in this study are available upon request by contact with the corresponding author.
